# Electronic cigarettes may not be a “safer alternative” of conventional cigarettes during pregnancy: evidence from the nationally representative PRAMS data

**DOI:** 10.1186/s12884-020-03247-6

**Published:** 2020-09-23

**Authors:** Sooyong Kim, Sanda Cristina Oancea

**Affiliations:** 1 Behavioral Sciences Group, Sanford Research, 2301 East 60th St N, Sioux Falls, SD 57104 USA; 2 Present address: Pinney Associates, Inc, 201 North Craig Street, Suite 320, Pittsburgh, PA 15213 USA; 3 Department of Population Health , University of North Dakota School of Medicine & Health Sciences, Grand Forks , ND 58202 USA

**Keywords:** Electronic cigarette, Prenatal tobacco use, Adverse birth outcomes, PRAMS

## Abstract

**Background:**

Conventional cigarette (CC) smoking is one of the most preventable causes of adverse birth outcomes. Although electronic cigarettes (ECs) are considered to be safer than CCs during pregnancy, the evidence is yet to be presented. This study examines the effects of prenatal EC use on neonatal birth outcomes compared to those of CC smokers and complete tobacco abstainers.

**Methods:**

Data was extracted from 55,251 pregnant women who participated in the Phase 8 survey of the Pregnancy Risk Assessment Monitoring System between 2016 and 2018. Participants were classified into three groups based on their smoking behaviors in the third trimester: complete tobacco abstinence, exclusive CC smoking, or exclusive EC use. Adverse outcomes included infants being small-for-gestational-age (SGA), having low birthweight (LBW), and being born at preterm. EC users were matched to complete abstainers and CC smokers who share the same baseline characteristics in race/ethnicity, age, educational attainment, income, prenatal care adequacy, and first- and second-trimester CC smoking statuses. The association between EC use and adverse birth outcomes were examined by survey-weighted logistic regression analyses in the matched population.

**Results:**

Among participants, 1.0% of women reported having used ECs during the third trimester, 60% of which reported using ECs exclusively. Neonates of EC users were significantly more likely to be SGA (OR 1.76; 95% CI 1.04, 2.96), have LBW (OR 1.53; 95% CI 1.06, 2.22), or be born preterm (OR 1.86; 95% CI 1.11, 3.12) compared to tobacco abstainers. However, odds of EC users’ pregnancies resulting in SGA (OR 0.67; 95% CI 0.30, 1.47), LBW (OR 0.71; 95% CI 0.37, 1.37), or preterm birth (OR 1.06; 95% CI 0.46, 2.48) were not significantly lower than those of CC smokers.

**Conclusions:**

Even after accounting for shared risk factors between prenatal tobacco use and adverse birth outcomes, EC use remains an independent risk factor for neonatal complications and is not a safer alternative to CC smoking during pregnancy. Until further research is completed, all pregnant women are encouraged to abstain from all tobacco products including ECs.

## Background

Prenatal smoking is one of the most important preventable causes of neonatal morbidity and mortality [1]. Smoking during pregnancy is not only associated with immediate, adverse birth outcomes–such as intrauterine growth restriction [1–4], preterm birth [4, 5], low birthweight (LBW) [3–8], and perinatal mortality [1, 4–6] but also known to affect the long-term physical [9, 10] and behavioral [11–13] health of children. Due to prenatal smoking’s detrimental effects and modifiable nature of the behavior, smoking cessation in pregnant women has been a major focus of intervention in the field of maternal-child health [14].

Studies show that, as a result of multiple public health interventions implemented to increase awareness and help women quit smoking, most pregnant smokers are aware of the harmful effects of prenatal smoking [15] and express a strong desire to stop smoking [16]. However, only 18–25% of them succeed at complete abstinence [17], resulting in 7.2% of pregnant women still being smokers during their pregnancies in 2016 [18]. While traditional cigarettes remain a significant concern, newly-emerging noncigarette tobacco products introduce another challenge in tobacco control among pregnant women.

An electronic cigarette (EC) is a battery-powered device that is similar in shape to traditional tobacco products, such as conventional cigarettes (CCs), cigars, or pipes. ECs vaporize a solution of nicotine, marijuana, flavors, or other chemicals for inhalation [19]. First introduced in the US in 2006, ECs have become extremely popular, especially among adolescents [20]. While recent reports on acute lung injury associated with EC use is extremely concerning and warrants immediate attention [21], its long-term health effects, including in utero exposure, is yet to be elucidated [22]. Investigating the effects of EC use on birth outcomes is a critical public health issue for multiple reasons. First, those who initially adopted ECs as teenagers have now reached reproductive age. Secondly, many pregnant women perceive that using ECs is safer than smoking CCs and will help them to quit smoking [23–27], even though scientific evidence supporting such an argument is scarce.

With the first-generation EC users reaching reproductive age, a common perception of women on ECs being a safer alternative, and their desire to quit smoking during pregnancy, recent studies have shown a concerning pattern of EC use in pregnant women in the US. Specifically, more than 10% of pregnant women have ever used ECs [24, 28], and between 1.5% and 5% of women kept using them during their pregnancies [28, 29]. However, in contrast to common perceptions among pregnant women, it is the consensus of scientific literature that any amount of prenatal nicotine exposure is dangerous to the developing fetus [30–32]. Unfortunately, due to EC’s relatively recent emergence and the unavailability of representative data, the effect of prenatal EC use on maternal and neonatal outcomes has rarely been investigated [19, 33]. While Wang et al. recently have presented one of the first epidemiologic evidence on prenatal EC use and its effect on birth outcomes [33], follow-up studies with a larger sample and more sophisticated control of shared sociodemographic risk factors have been called for.

The objective of this study is to investigate the comparative effects of pregnant women’s EC use, CC smoking, and complete tobacco abstinence on neonatal outcomes with propensity matching. The findings of this research will serve as compelling evidence for public health interventions for tobacco control. The findings will also help set obstetric, pediatric practice guidelines on counseling pregnant smokers who are current EC users or interested in using ECs as a smoking cessation aid.

## Methods

### Hypotheses

This study examines the two following hypotheses. First, it is hypothesized that exclusive EC use in the third trimester significantly affects birth outcomes–such as infants being small-for-gestational-age (SGA), having low birthweight (LBW), and being born at preterm– compared to complete abstinence from tobacco. The third trimester was selected because the last three months of pregnancy are a crucial period for the fetus to gain weight and be born as a full-term [34], and the most common adverse effects of prenatal tobacco exposure concern birthweight and preterm birth [1–8]. Since several studies have reported that the adverse effects of smoking on birthweight and gestational age do not occur until the third trimester [35–37], demonstrating the effects of EC use in the third trimester will have a significant clinical implication for preventing such adverse birth outcomes.

The second hypothesis of this research is, in opposition to the common belief of pregnant women [23–27], that the risks of using ECs are not significantly lower than those of smoking CCs. This hypothesis is based on current evidence that ECs also contain a considerable amount of nicotine [38] and produce highly oxidizing free radicals [39], which are the components of CCs that are responsible for its adverse birth effects [40, 41].

### Sample

**Fig. 1:**
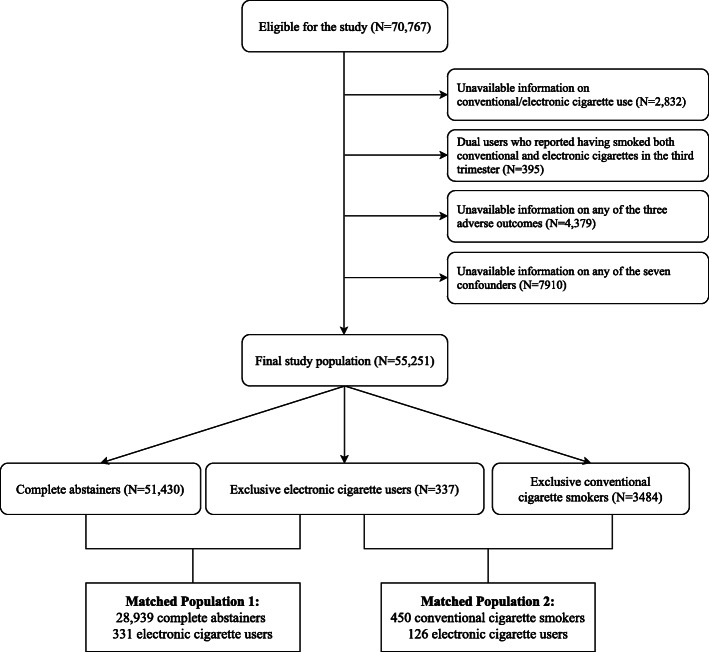
Selection of the final study sample

Data were extracted from the Phase 8 survey of the Pregnancy Risk Assessment Monitoring System (PRAMS), collected between 2016 and 2018. The study design and the methodology have been detailed in a previous publication [42]. The PRAMS is a nationally representative surveillance program in the U.S. that is conducted to monitor women’s experiences and attitudes regarding their pregnancies. By interviewing women who recently gave live birth, the PRAMS provides a unique opportunity to understand the impact of maternal behavior on infantile morbidity and mortality. To date, Phase 8 of the PRAMS is the only representative survey that includes data on EC use in pregnant women and the birth outcomes of their infants. Among the 47 US states, areas and territories that participate in the PRAMS, data from 70,767 women in 36 states, New York City, and Puerto Rico were available at the time this research was conducted. Final analyses were performed on 53,971 participants who met this study’s inclusion and exclusion criteria of this study (Fig. 1, upper half). Further matching process is described below. The PRAMS data was provided by the Center of Disease Control and Prevention upon the approval of the research proposal author submitted prior to this study. After the acquisition of the data, this research has been reviewed and approved by the Institutional Review Board of the University of North Dakota.

### Predictor: smoking behaviors in the third trimester

Participants were asked how often they used ECs or other electronic nicotine products during the last three months of their pregnancies. Electronic nicotine products other than ECs include various devices, such as e-hookahs, mods, vapes, and vape pens [19]. However, all of them are similar in operation and components [19]. Therefore, the term “ECs” will be used throughout this article to represent all types of electronic nicotine products, including ECs themselves. EC use in the first and second trimester was not collected in the PRAMS survey. Five options were given, based on the number of days the respondent used the ECs: more than once a day, once a day, two to six days a week, one day a week or less or no use of any of such products. The variable was dichotomized into two levels: having used ECs in the third trimester or never having done so. In contrast, data for CC smoking were collected for all trimesters and responses were collected in the number of cigarettes smoked per day during each trimester. Third trimester CC smoking was also dichotomized into two levels: either having smoked CCs in the third trimester or never having done so.

Respondents were categorized into four groups based on their smoking behaviors in the third trimester. Those who did not use any tobacco products–specifically, those who had never smoked CCs *and* never used ECs in the third trimester–were defined as “complete abstainers”. Participants who reported having smoked CCs but have never having used ECs in their third trimesters were defined as “exclusive CC smokers”. Women who used ECs but never smoked CCs in their third trimesters were defined as “exclusive EC users”. Lastly, those who reported having smoked CCs *and* used ECs in their third trimesters were defined as “dual-users”.

Dual-users were excluded from the final study sample for the following reasons. First, dual-users encompass a highly heterogeneous group of tobacco users with variable patterns of CC and EC use, [33] complicating the interpretation even if associations were found to be significant. Moreover, preliminary analyses showed that the daily number of CCs smoked among dual-users was not significantly different from that of exclusive CC smokers (*p* = .291), which was also a ground for assuming mixed-effects with limited interpretability.

### Outcome: SGA, LBW, preterm birth

Three adverse birth outcomes were investigated for possible associations with smoking behaviors in the third trimester: (1) SGA, (2) LBW and (3) preterm birth. SGA was defined as birthweight lower than the 10th percentile among neonates born at the same gestational age [43]. Birthweights less than 2,500 gm were considered LBWs [44], and birth before the completion of 37 weeks of pregnancy was defined as preterm [45].

### Risk factors for adverse birth outcomes

Seven maternal, familial, obstetrical factors are known to affect birth outcomes were defined for the subsequent matching process (described below).

Maternal demographics included race/ethnicity (categorized into White, Black, Hispanic, Others); age (collected as 17 or younger, 18–19, 20–24, 25–29, 30–34, 35–39, or 40 or older; recoded by the median age for each category); and educational attainment (collected as 0–8 years, 9–11 years, 12 years, 13–15 years, 16 years or more; collapsed to less than high school, high school graduates, some college education, and bachelor’s degree or more). The first and second trimester CC smoking was dichotomized into two variables representing whether the participants have ever smoked during each respective trimester.

Family factors included yearly income (recoded to four levels ranging from less than $25,000, $25,000-$50,000, $50,000-$75,000, to more than $75,000).

Obstetrical factors included prenatal care appropriateness (collected as inadequate, intermediate, adequate, or adequate plus, as defined as in the Kotelchuck Index [46]; recoded to either appropriate or inappropriate).

### Matching between the EC user group and the reference group

Previous studies have shown that nonsmokers have significantly different characteristics from tobacco users [47]. Even among tobacco users, EC users and CC smokers also show significant differences in their baseline characteristics [48], evidenced by the significant differences in covariates among the three tobacco user groups (see Table 1 below). As complete abstainers, EC users and CC smokers are vastly and systematically different, simply adjusting for covariates in conventional regression models leaves residual confounding where the effect of shared risk factors and prenatal EC use cannot be isolated [49]. In fact, the imbalance in baseline characteristics and lack of finer control of them were one of the limitations acknowledged by Wang et al. in their recent studies on prenatal EC use [33].

**Table 1:** Descriptive statistics of the sample

	Complete abstainers (*N* = 51,430)	Exclusive EC users (*N* = 337)	Exclusive CC smokers (*N* = 3,484)	Total (*N* = 55,251)
Race/ Ethnicity*				
White	24,160 (46.98%)	209 (62.02%)	2,162 (62.06%)	26,531 (48.02%)
Black	9,220 (17.93%)	43 (12.76%)	595 (17.08%)	9,858 (17.84%)
Hispanic	10,371 (20.17%)	45 (13.35%)	200 (5.74%)	10,616 (19.21%)
Others	7,679 (14.93%)	40 (11.87%)	527 (15.13%)	8246 (14.92%)
Age*	27 (27–32)	27 (22–32)	27 (22–32)	27 (27–32)
Educational attainment*				
Less than HS	5,642 (10.97%)	59 (17.51%)	870 (24.97%)	6,571 (11.89%)
HS graduate	11,520 (22.40%)	123 (36.50%)	1,444 (41.45%)	13,087 (23.69%)
Some college	15,086 (29.33%)	122 (36.20%)	1,064 (30.54%)	16,272 (29.45%)
Bachelor or more	19,182 (37.30%)	33 (9.79%)	106 (3.04%)	19,321 (34.97%)
Familial income*				
<$25 k	18,344 (35.67%)	215 (63.80%)	2,517 (72.25%)	21,076 (38.15%)
$25 k-$50 k	10,459 (20.34%)	68 (20.18%)	614 (17.62%)	11,141 (20.16%)
$50 k-$75 k	7,078 (13.76%)	29 (8.61%)	231 (6.63%)	7,338 (13.28%)
>$75 k	15,549 (30.23%)	25 (7.42%)	122 (3.50%)	15,696 (28.41%)
Adequate prenatal care*	40.269 (78.3%)	236 (70.03%)	2,294 (65.84%)	42,799 (77.46%)
First trimester CC smoking*	1,011 (1.97%)	46 (13.65%)	3,396 (97.47%)	4,453 (8.06%)
Second trimester CC smoking*	302 (0.59%)	20 (5.94%)	3,397 (97.50%)	3,719 (6.73%)

*EC* Electronic cigarette, *CC* Conventional cigarette, *HS* High school

Asterisk(*) represents a statistically significant difference between three groups for each respective maternal characteristic, calculated from either weighted chi-squared tests for categorical variables or weighted, unadjusted linear regression analyses for continuous variables.

Note: descriptive statistics are given as frquency (percentage) for categorical variables and median (interquartile range) for continuous variables.

To more comprehensively account for the fundamental differences in risk factors, propensity matching was induced between exclusive EC users and two reference groups (complete abstainers, exclusive CC smokers) based on the seven covariates described above. A “control” group was created with either complete abstainers or CC smokers, and the “treatment” group was defined as EC users. Exact propensity matching was conducted using the “MatchIt” package [50] to match each observation in the treatment group (an EC user) to all possible observations in the control group (complete abstainers or CC smokers) that share exact characteristics of seven covariates. Exact matching yielded (1) 28,939 complete abstainers and 331 EC users and (2) 450 CC smokers and 126 EC users for each analysis (see Fig. 1, lower half). The minimum sample size for logistic regression was calculated using the “powerMediation” package [51]. A minimum of 19,855 and 502 observations were required for the first (comparison between EC users and complete abstainers) and the second model (between EC users and CC smokers), which were well exceeded by the numbers of matched populations. The use of exact matching, rather than nearest-neighbor or full matching, did not require further diagnostic measures for ensuring the balance in covariates between two groups.

### Analyses

Survey-weighted logistic regression analyses were performed in the matched populations to demonstrate the effects of EC use on adverse birth outcomes compared to abstinence from tobacco and CC smoking in the third trimester. Three outcomes–a) SGA, b) LBW, c) preterm birth– were tested in two matched populations–1) EC users and complete abstainers and 2) EC users and CC smokers, totaled six analyses. Since the analyses were conducted in the matched populations which are already exactly the same in their baseline characteristics, no further adjustments for covariates were made.

The “survey” package [52] was used to calculate survey-weighted descriptive statistics and conduct survey-weighted analyses. Data management and analyses were conducted using R software [53].

## Results

The final study sample prior to matching consisted of 55,251 women who met the inclusion and exclusion criteria of this study. The baseline characteristics are described in Table 1 with the study sample before matching was conducted. The vast majority of the participants were complete abstainers (weighted percentage (WP) = 94.80%), followed by exclusive CC smokers (WP = 4.62%), and exclusive EC users (WP = 0.58%). Each group showed significantly different distributions in the maternal and obstetric characteristics included in the final analyses.

Compared to complete abstainers, EC users were likely to be White, younger, and less educated, to have lower incomes, and to have smoked during the prior trimesters (all *p* < .001). However, the rate of having adequate prenatal care was not significantly different between EC users and complete abstainers (*p* > .05). Compared to CC smokers, EC users were likely to be younger, more educated, to have higher income, to receive appropriate prenatal care, and to have not smoked during the previous trimesters (all *p* < .001).

The weighted percentages of adverse outcomes investigated in this study were generally much higher among tobacco users (Table 2). However, the rates of pregnancies of EC users resulting in SGA (WP = 14.00%), LBW (WP = 7.34%), or preterm birth (WP = 11.74%) were not significantly higher than complete abstainers (WP = 8.62% for SGA / 5.37% for LBW / 7.15% preterm), even though the overlap between the confidence intervals were minimal. Similarly, rates of neonates being born SGA and preterm were not significantly different among EC users (WP = 14.00% for SGA / 11.74% for preterm) compared to those of CC smokers (WP = 21.86% for SGA / 10.57% for preterm). However, the rate of infants having LBW was significantly lower among EC users (WP = 7.34%) than CC smokers (WP = 11.96%).

**Table 2:** Descriptive statistics of adverse birth outcomes

		Complete abstainers (*N* = 51,430)	Exclusive EC users (*N* = 337)	Exclusive CC smokers (*N* = 3,484)	Total (*N* = 55,251)
**SGA**	N	6,981	66	1,017	8,064
	WP	8.62%	14.00%	21.86%	9.26%
	95% CI	8.29–8.96%	8.88–21.38%	19.80–24.07%	8.93–9.60%
**LBW**	N	9,073	86	1,149	10,308
	WP	5.37%	7.34%	11.96%	5.68%
	95% CI	5.18–5.57%	5.22–10.21%	10.81–13.22%	5.50–5.88%
**Preterm**	N	7,674	66	752	8,492
	WP	7.15%	11.74%	10.57%	7.33%
	95% CI	6.86–7.44%	7.37–18.20%	9.26–12.05%	7.05–7.62%

*EC* Electronic cigarette, *CC* Conventional cigarette, *SGA* Small-for-gestational-age, *LBW* Low birthweight

Note: Statistics are given as unweighted frequency (N), survey-weighted percentage, (WP) and weighted 95% confidence intervals (95% CI).

Results from logistic regression analyses on the matched population are presented in Table 3. The general tendency of adverse outcomes being more common among non-abstainers persisted, even after matching EC users and complete abstainers based on their baseline characteristics known to affect birth outcomes (Table 3, row 1). The odds of having adverse outcomes were significantly and consistently higher among EC users compared to complete abstainers. Specifically, neonates of women who used ECs during their third trimesters had significantly higher odds of being born SGA (OR 1.76; 95% CI 1.04, 2.96), having LBW (OR 1.53; 95% CI 1.06, 2.22) and being born preterm (OR 1.86; 95% CI 1.11, 3.12).

**Table 3:** Results from weighted logistic regression after matching: effects of prenatal electronic cigarette use on adverse outcomes compared to different reference groups

Reference group	SGA		LBW		Preterm birth	
	**OR**	**95% CI**	**OR**	**95% CI**	**OR**	**95% CI**
**Complete abstainers**	1.76	1.04–2.96*	1.53	1.06–2.22*	1.86	1.11–3.12*
**CC smokers**	0.67	0.30–1.47	0.71	0.37–1.37	1.06	0.46–2.48

*: *p*<0.05

*OR* Weighted, adjusted odds ratio, *CI* Confidence interval, *CC* Conventional cigarette, *SGA* Small-for-gestational-age, *LBW* Low birthweight

Note: Statistics represent the Odds Ratio (95% Confidence Interval (CI)). EC users were exact-matched to the respective reference group based on the maternal race, age, educational attainment, family income, prenatal care adequacy, and conventional cigarette smoking status of the first and second trimester.

Using ECs during the third trimester, however, failed to show significantly lower odds of having adverse birth outcomes compared to smoking CCs (Table 3, row 2). Between CC smokers and EC users, there were no significant differences in the odds of neonates being SGA (OR 0.67; 95% CI 0.30, 1.47), having LBW (OR 0.71; 95% CI 0.37, 1.37) or being born preterm (OR 1.06; 95% CI 0.46, 2.48).

## Discussion

The objective of this study was to investigate the comparative effect of prenatal EC use on birth outcomes compared to complete tobacco abstinence and CC smoking. With early EC adopters reaching reproductive age and “harm reduction” advertising strategies changing the perceptions more favorably [25, 27], 1.0% of pregnant women reported using ECs during their third trimesters and 60% of them were exclusive EC users without smoking CCs. However, the adverse effect of EC use on birth outcomes was consistent with the two hypotheses of this study. First, EC use in the third trimester has shown significantly positive associations with all three outcomes – SGA, LBW, and preterm infants. Moreover, in opposition to the common belief that ECs are safer than CCs, using ECs instead of smoking CCs showed hardly any benefit for reducing adverse neonatal outcomes. Collectively, the results indicate that not only does prenatal EC use have likely adverse effects on fetuses, but using ECs is also not any safer than smoking CCs.

The present study is in a partial agreement with the previous work by Wang et al. [33], in which authors demonstrated increased odds of SGA but no significant differences in the likelihood of preterm birth between exclusive EC users and complete abstainers. Although current study and Wang et al.’s work share several major components in common, such as the use of Phase 8 PRAMS data, definition of exclusive CC/EC use, and outcomes of interests, this study addresses the limitations acknowledged in Wang et al.’s work and advances the understanding of the literature in a number of ways. First, this study utilizes a much larger sample, which yields more tobacco users and therefore, much narrower confidence intervals. Secondly, the propensity score matching used in this study allows more sophisticated adjustment for socioeconomic risk factors that are shared between prenatal tobacco use and adverse birth outcomes. Furthermore, a comparison of birth outcomes between EC users and CC smokers provides practical evidence for clinicians and pregnant women who wish to gauge risk and benefit between CCs and ECs.

Lastly, the results of this study are in line with the extant literature that ECs are not safe during pregnancy [31, 54–59]. Although most of the current evidence on ECs’ effects on developing fetuses is either extrapolated from the previous research on nicotine exposure in utero [31, 54, 55] or drawn from on animal experiments in the lab settings [56–59], transplacental delivery of nicotine is considered key to the pathophysiology of ECs’ effects on the fetus [32, 59]. Given that 99% of EC products sold in the US contained nicotine [60], it is plausible to assume that the nicotine from the EC is conveyed to the fetus after absorbed in the mother’s system, causing multiple adverse outcomes, including, but not limited to, the infant being SGA, having LBW, or being born at preterm.

The findings of this study contradict the common perception among pregnant smokers that ECs can be a safer alternative to CCs [23–27]. Although the nicotine delivery of ECs depends on a number of factors, such as the make of the device, the level of nicotine in the liquid, and the puffing behavior of individuals, the plasma concentration of nicotine after EC use can be as high as that after CC smoking [61]. The fact that ECs do not contain tobacco and do not produce tar or carbon monoxide [62], does not mean they are safe for the general population [19, 22], let alone pregnant women and their unborn fetuses.

### Limitations

This study should be interpreted while contemplating some limitations. The most important factors to consider are the way EC use was measured and the characteristics of the behavior which this study was not able to account for. First, the findings of this study solely concern the EC use in the third trimester, without accounting for EC use in the first and second trimesters. Although earlier trimesters are more closely related to birth defects and stillbirth than to birthweight and gestational age [63] and CC smoking statuses during these trimesters were accounted for, the effects of exposure to ECs during the period of organogenesis should not be underestimated.

Secondly, due to the limited number of participants who exclusively used ECs in their third trimesters, those who reported using ECs less than once a week were dichotomized into the same group with daily users. While there is a strong possibility of underreporting due to the recall-based, self-reported nature of the PRAMS data and the stigma of using ECs during pregnancy [26], this study cannot test any dose-dependent relationship due to this simplification.

There are also factors that play a major role in determining blood chemical levels but could not be taken into consideration in this study. For example, e-liquid can contain multiple chemicals, such as nicotine, marijuana, and flavors. The composition is also highly personalized, ranging from nicotine-free to 36 mg/mL of nicotine. Furthermore, the level of nicotine in the blood, which is transmitted to the fetus, can vary significantly from the labeled level of the e-liquid due to individual puffing behaviors [64]. Taken altogether, it is critical to have a comprehensive understanding of EC use behavior to estimate the maternal blood level of toxicants, above and beyond the simple frequency of use.

Lastly, other limitations include the possible effects of unmeasured confounders that were not accounted for the matching process and cross-sectional nature of the PRAMS data that limits causal inferences. Based on the limitations of this study, further research with large longitudinal data that capture the dynamics of EC use behavior is strongly recognized.

### Strengths

This study has several strengths as well. Most importantly, the findings of this study have major clinical significance as an epidemiological study that demonstrates the effects of prenatal EC use on birth outcomes in humans. This research contains the evidence that has been greatly called for, given the increasing popularity of ECs and the aggressive marketing strategies of tobacco companies. Although the limitations necessitate further research, this study opens up a critical discussion for managing prenatal smoking in the era of novel tobacco products. Moreover, this research is conducted based on the PRAMS data, which includes large representative samples from 36 US states, New York City and Puerto Rico. Therefore, the study’s conclusions are more readily generalizable than those of studies relying on a limited number of observations or convenience sampling that are prone to selection bias.

## Conclusions

Tobacco use during pregnancy is the most important preventable cause of adverse birth outcomes while ECs present additional challenges. This study suggests the significant adverse effects associated with EC use on birth outcomes, as well as non-superiority over CC smoking. In conclusion, this study fails to support the common perception of pregnant women who considers ECs to be safer alternatives to CCs during pregnancy and raises concerns about their safety for the unborn fetus. While further research is required to elucidate the true effects of prenatal EC use, pregnant smokers should be counseled regarding the possible adverse effects of ECs and advised to abstain from any tobacco product including ECs until proven otherwise.

## Data Availability

The PRAMS data used in this study was provided from the PRAMS working groups and the Centers for Disease Control and Prevention and can be accessed upon request.

## Abbreviations

PRAMS: Pregnancy Risk Assessment Monitoring System
EC: Electronic cigarette
CC: Conventional cigarette
SGA: Small-for-gestational-age
LBW: Low birthweight
